# Hearts of *Dystonia musculorum* Mice Display Normal Morphological and Histological Features but Show Signs of Cardiac Stress

**DOI:** 10.1371/journal.pone.0009465

**Published:** 2010-03-01

**Authors:** Justin G. Boyer, Kunal Bhanot, Rashmi Kothary, Céline Boudreau-Larivière

**Affiliations:** 1 School of Human Kinetics, Laurentian University, Sudbury, Ontario, Canada; 2 Department of Biology, Laurentian University, Sudbury, Ontario, Canada; 3 Biomolecular Sciences Program, Laurentian University, Sudbury, Ontario, Canada; 4 Regenerative Medicine Program, Ottawa Hospital Research Institute, Ottawa, Ontario, Canada; 5 Department of Cellular and Molecular Medicine, University of Ottawa, Ottawa, Ontario, Canada; McMaster University, Canada

## Abstract

Dystonin is a giant cytoskeletal protein belonging to the plakin protein family and is believed to crosslink the major filament systems in contractile cells. Previous work has demonstrated skeletal muscle defects in dystonin-deficient *dystonia musculorum* (*dt*) mice. In this study, we show that the dystonin muscle isoform is localized at the Z-disc, the H zone, the sarcolemma and intercalated discs in cardiac tissue. Based on this localization pattern, we tested whether dystonin-deficiency leads to structural defects in cardiac muscle. Desmin intermediate filament, microfilament, and microtubule subcellular organization appeared normal in *dt* hearts. Nevertheless, increased transcript levels of atrial natriuretic factor (ANF, 66%) β-myosin heavy chain (beta-MHC, 95%) and decreased levels of sarcoplasmic reticulum calcium pump isoform 2A (SERCA2a, 26%), all signs of cardiac muscle stress, were noted in *dt* hearts. Hearts from two-week old *dt* mice were assessed for the presence of morphological and histological alterations. Heart to body weight ratios as well as left ventricular wall thickness and left chamber volume measurements were similar between *dt* and wild-type control mice. Hearts from *dt* mice also displayed no signs of fibrosis or calcification. Taken together, our data provide new insights into the intricate structure of the sarcomere by situating dystonin in cardiac muscle fibers and suggest that dystonin does not significantly influence the structural organization of cardiac muscle fibers during early postnatal development.

## Introduction

Plakin crosslinking proteins such as dystonin and plectin have been implicated in regulating the cytoskeletal organization and function of muscle (recently reviewed in [1], [2]). While a number of recent studies have further defined the role of plectin in muscle tissue [3], [4], [5], [6], much less progress has been made in elucidating the functions of dystonin in contractile cells.

Several different dystonin isoforms are produced through alternative splicing of the dystonin gene [7], [8], [9]. Dystonin isoforms are expressed in a tissue-specific manner and include an epithelial isoform (dystonin-e) [10], neuronal isoforms (dystonin-a) [8], [11], [12], as well as muscle isoforms (dystonin-b) [8]. The muscle and neuronal isoforms can be further characterized by three unique N-terminal regions (dystonin-b1, b2, b3/a1, a2, a3) that influence the subcellular localization of these proteins [13], [14], [15], [16]. The dystonin-b muscle isoforms are the largest (834 kDa) and consist of several domains: an N-terminal actin-binding domain (ABD), a plakin domain, a spectrin repeat containing rod domain, a centrally located intermediate filament binding domain (IFBD2) and a microtubule-binding domain (MTBD) at the C-terminus [8].

The *dystonia musculorum (dt)* mouse mutant has been studied as a model of sensory neuropathy since its initial identification [17], [18]. Several allelic variants of *dt* exist in which mutations of the dystonin gene result in a dramatic reduction and virtual loss of dystonin gene expression [19]. In the *dt^Tg4^* mouse model [20], intrinsic skeletal muscle defects have previously been reported [21]. Specifically, skeletal muscles from the *dt^Tg4^* mice have thick and poorly defined Z- discs and display a reduction in sarcomere length as well as abnormal mitochondrial clumping under the sarcolemma [21]. Furthermore, the *dt^Tg4^* skeletal muscles are weak and fragile. These skeletal muscle defects likely contribute to the limb incoordination phenotype displayed by these mice. Dystonin appears to play a more critical role in maintaining the stability of the cytoarchitecture in skeletal muscle fibers, rather than in the establishment of the cytoskeletal networks during muscle formation and development [21]. This notion is further supported by primary myogenic cell culture experiments where it was shown that the proliferation and differentiation potential of *dt^Tg4^* myogenic cells is similar to that of wild-type (wt) cells [22]. Collectively, these findings support the idea that dystonin maintains the structural integrity of skeletal muscle cells although the precise cellular mechanisms by which it does so, has not been fully described [21], [23]. Dystonin is highly expressed in cardiac muscle [8] and yet very little is known about the role of this molecule in heart tissue. Given the apparent function of dystonin in skeletal muscle cells, it is reasonable to expect that this crosslinking protein would have a key function in maintaining the structural integrity of cardiac tissue. In the present study, we show using a muscle isoform-specific dystonin antibody, that dystonin is localized at the Z-disc and H zone in cardiac muscle. We assessed the expression of genes routinely used as early indicators of cardiac myopathy, particularly cardiac hypertrophy and show that the expression profile of these markers in dystonin-deficient in comparison to wt hearts is suggestive of early signs of cardiac myopathy. However, our analysis did not reveal any morphological defects in early development which may be attributed in part to the young age of the animals.

## Materials and Methods

### Ethics Statement

The mice were cared for according to the Canadian Council on Animal Care (CCAC) guidelines. Ethical approval for experiments conducted was obtained from the Laurentian University Animal Care Committee.

### Mice and Tissue Excision

The *dystonia musculorum* Tg4 (*dt^Tg4^*) line of transgenic mice used in this study is from the CD1 strain background and has an insertional mutation within the dystonin gene [11], [20]. The mice were housed at the Laurentian University Animal Care Facility and cared for according to the Canadian Council on Animal Care (CCAC) guidelines.

Postnatal day 14 animals were anaesthetized with ketamine/rompun (0.1 mg/g, 0.01 mg/g) delivered by intraperitoneal injection before excising the tissues. The endpoint for the study was 14 days after birth as most *dt^Tg4^* mice do not survive past two weeks of age. The hearts used for morphological analyses were first injected with potassium chloride (KCl, 100 mM) to ensure each heart was stopped in diastole. Hearts were either flash frozen or fixed and then embedded in Histo Prep (Fisher Scientific International Inc., Hampton, NH) and frozen in pre-cooled isopentane.

### RNA Extraction and Reverse-Transcription Quantitative Polymerase Chain Reaction

Total RNA was isolated from the flash frozen hearts using the RNeasy kit as per the manufacturer's protocol (Qiagen). RNA (50 ng) was reverse transcribed using Moloney Murine Leukemia Virus Reverse Transcriptase (MMLV RT) (200 U/µl) (Invitrogen) and random hexamer (2.5 µM) primers. A negative control sample comprised of RNase/DNase free water in lieu of RNA was prepared and processed in parallel to rule out contamination.

Quantitative polymerase chain reaction (QPCR) (MJ Research) was performed in triplicate for each sample using primer pairs as detailed in Table 1. QPCR reactions contained 5 µl of cDNA, 12.5 µl of 2X iQ SYBR Green Supermix (Bio-Rad), 7.3 µl RNase/DNase-free water and 0.1 µl of each primer (200 nM). Each reaction was heated to 94°C for 3 min followed by 40 cycles with denaturation at 94°C for 1 min, primer pair-specific annealing temperature for 1 min and extension at 72°C for 1 min. A melting curve analysis was performed at the end of each experiment. The PCR product sizes were verified by resolving samples on agarose gels and PCR products were sequenced to confirm the identity of each target. The gene expression data were extrapolated from standard curves generated in parallel using known amounts of input RNA ranging from 200 ng to 3 ng. Gene expression data for each target were normalized to rRNA S12 (internal control).

**Table 1:** Primers used for QPCR experiments.

Targets	Sense primer 5′-3′	Antisense primer 5′-3′
ANF	GAGTGGGCAGAGACAGCAAA	CAGGTGAGACCCGAGGTTAG
BNF	CATGGATCTCCTGAAGGTGC	CAACACCGTCAAACACGAGG
SERCA2a	GAATCTGTCTCCGTCATCAAGC	GATTCGTCCACCACTACTGTCGTC
beta-MHC	GAAGATGCGGCGGGACCTGG	GAACGCTGCGTCTCCTCGGC
PLB	CGATCACCGAAGCCAAGGTCTCC	CGGTGCGTTGCTTCCCCCAT
S12 rRNA	GGAAGGCATAGCTGCTGG	CCTCGATGACATCCTTGG
MACF	GGGCCTGATCTGGACCATTATC	AAGTGCCTTTAGTTCAACAGGG
Plectin	CAAGGCTCAAAGGCACATCAG	CAGAAACTGGCACCATAGGA
Dp	TGGGTACCTGCCAAGATGT	TGAGCTCGTTGATTTTCACG

ANF, atrial natriuretic factor; BNF, b-type natriuretic factor; SERCA2a, sarcoplasmic reticulum calcium pump isoform 2A; beta-MHC, beta-myosin heavy chain; PLB, phospholamban; S12 rRNA, ribosomal RNA; MACF, microtubule-actin crosslinking factor; Dp, desmoplakin.

### Morphologic and Histological Analyses of Heart Tissue

Heart weight/body weight (h.w./b.w.) ratios were determined by weighing the mice and hearts using a Denver Instrument APX-602 scale and a Sartorius ISO 9001 analytical scale respectively. To assess the left ventricular wall thickness and chamber area, tissue sections at a thickness of 8 µm were collected from the same area of frozen wt and *dt* hearts using a Leica CM 3050 S cryostat set at −22°C. The muscle sections were stained with hematoxylin and eosin using a standard staining procedure then visualized using a Zeiss Axiovert 200 M fluorescent inverted deconvolution microscope and photographed using an AxioCam HR camera and the Axio Vision 4.2 computer software program. The cardiac wall thickness and the area of the left ventricle chamber were determined using the software measurement tool. In parallel experiments, muscle sections from wt and *dt* hearts were stained for the presence of abnormal calcium deposits and fibrosis using the Von Kossa and Masson Trichrome staining methods respectively.

### Generation of an Antibody Specific to the Muscle Isoform of Dystonin

To generate an antibody specific to the muscle isoform of dystonin, a pET30a plasmid expressing 6x his-tags at both the N-terminal and C-terminal end of a short fragment encoding a region from the dystonin isoform-b was used. The fusion construct coded for the IFBD2 (bp 6980 – bp 8142 GenBank Accession NM134448.2), an isoform b specific region just downstream of the plakin domain. The his-tagged IFBD2 construct (his-IFBD2) (previously generated) was produced in soluble form using the bacterial protein expression protocol as described (Supplemental Information S1). A gradient pump (Bio-Rad) was used to pass 50 ml (5 mg/ml) of the soluble fraction from bacterial lysate containing the his-tagged fusion protein through a high-flow his-trap column (GE healthcare) as per the manufacturer's protocol. The eluate obtained was concentrated by placing it in dialysis tubing (porosity for MW 8000) and immersing it in a solution of poly-ethylene-glycol (MW 15000) (Sigma) for 3 hours at 4°C under gentle agitation. A total of 4 mg of concentrated protein was sent to Cedarlane Laboratories and was used to inoculate 2 chickens. Serum containing the antibody was used for biochemical analyses to determine specificity and efficacy of the final product.

### Immunoblotting Analysis

Protein extracts from wt and *dt* hearts were obtained by grinding the tissues in liquid nitrogen and adding the powder to ice-cold lysis buffer (150 mM NaCl, 10 mM Tris pH 7.5, 1 mM EDTA pH 7.0, 1 mM EGTA pH 9.0, 1% Triton-X 100, 0.5% Igepal CA-630) containing protease inhibitors (1 µg/ml Leupeptin, 1 µg/mlAprotinin, 5 µg/ml PMSF). Protein concentrations were determined using the Bradford method (Bio-Rad). Protein lysates were subjected to SDS- polyacrylamide gel electrophoresis and transferred from the gel to a PVDF membrane (Millipore) using a wet-transfer apparatus (Bio-Rad). Transfer efficiency was verified by staining the membrane with Ponceau S (Fisher). Antibodies targeting desmin (1∶400, mouse, Sigma), tubulin (1∶1333, mouse, Sigma), actin (1∶1000, mouse, Sigma) and his-epitope (1∶1000, mouse, Cell Signaling), and subsequently detected with a horseradish peroxidase-conjugated goat anti-mouse IgG (H+L) secondary antibody, were used to assess the levels of these cytoskeletal proteins. Immunodetection was performed using the enhanced chemiluminescent (ECL) kit (GE Life Sciences) according to the manufacturer's protocol and a gel documentation system (Alpha Innotech Corporation).

Densitometric analysis was performed using the FluroChem software (Alpha Innotech Corporation). The membranes were subsequently stained with EZBlue gel staining reagent (Sigma) then scanned using the GS-800 Imaging Densitometer (Bio-Rad) to determine total protein per lane which served as a control for loading variability.

### Immunofluorescence

Serial longitudinal (12 µm) sections from wt and *dt* hearts were incubated with antibodies targeting either, dystonin-b (1∶1000, chicken), desmin (diluted 1∶2, D3 cell supernatant, mouse, Developmental Studies Hybridoma Bank) or tubulin (diluted 1∶1000, mouse, Sigma). Nuclei were visualized with DAPI (Sigma) and phalloidin 546 (Invitrogen) was used to label actin. Furthermore, cardiac myosin heavy chain (1∶200, mouse, Abcam), α-actinin (1∶100, mouse, Abcam), vinculin (1∶100, mouse, Sigma) and desmoplakin (1∶20, mouse, Progen) antibodies were used to highlight sarcomeric, sarcolemmal and adhesion reference points. The Flag-tag antibody was diluted at 1∶1500 (mouse, Sigma) and the trans-Golgi network marker-38 (mouse, Acris) as well as the endoplasmic reticulum marker protein disulfide isomerase antibody (mouse, Abcam) were diluted at 1∶300. The cells were maintained in culture and transfected as described elsewhere [14]. Samples were fixed with 4% paraformaldehyde at room temperature and stained as previously described [13]. All images were acquired using a Zeiss confocal microscope (LSM 510 META DuoScan).

### Statistical Analysis

Student's t-test and one-way ANOVAs were used to establish the differences between *dt* and wt mice with regards to the following variables: h.w./b.w. ratios, left ventricular wall thickness and area, protein levels, and transcript levels of both the hypertrophic markers and plakins. All statistical analyses were performed using the SPSS software (version 12) and significance was set at the P<0.05.

## Results

### Generation and Characterization of an Antibody Against the Dystonin Muscle Isoform

**Figure 1:**
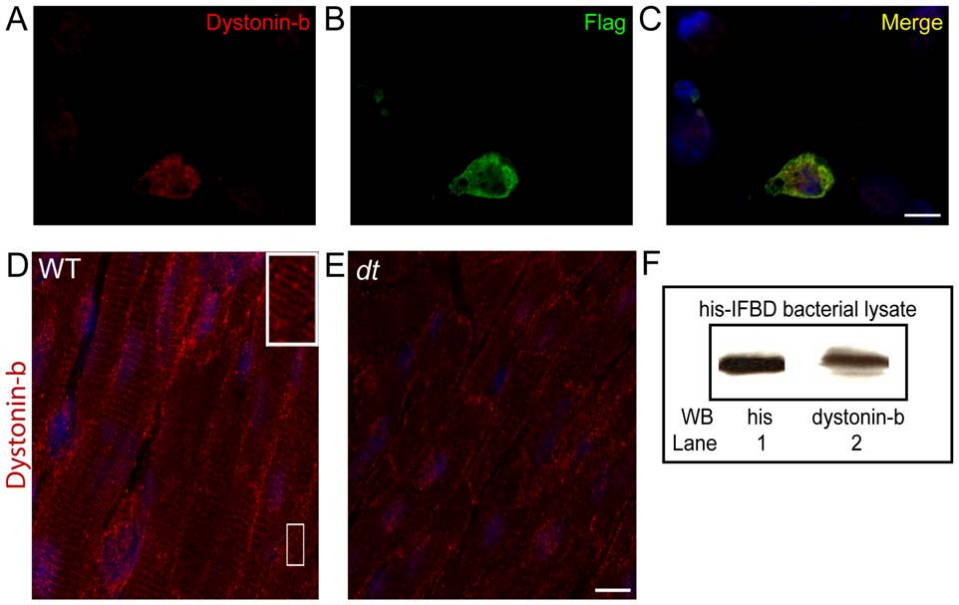
Dystonin-b antibody specificity and efficacy. Micrographs of a transfected Cos-1 cell double-stained with the dystonin-b antibody (A) and a flag-epitope antibody (B). Dystonin-b staining is specific to the flag-fusion protein expressing cell (C). Nuclei are visualized in blue. Longitudinal sections of cardiac muscle from age-matched wt and *dt* hearts were immunostained with dystonin-b antibody (D and E). A distinct striated pattern was observed in wt samples (D). In comparison, the signal is considerably attenuated in *dt* samples where faint striations were detected in very few fibers (E). Immunodetection analysis was performed on bacterially expressed his-IFBD protein used as the antigen for the generation of the dystonin-b antibody (F). Lane 1; protein band is detected with an anti-his antibody. Lane 2; a protein of similar size is detected with the dystonin-b antibody. Scale bar  = 10 µm indicated in panel C is also applicable to panels A and B. Scale bar  = 10 µm indicated in panel E is also applicable to panel D. N = 5 for cardiac tissue staining.

Immunoblot and immunofluorescence analyses were performed to determine the specificity of our dystonin-b antibody generated against the IFBD2 region within the muscle isoform of the protein. A western blot was performed on bacterially expressed his-IFBD2 fusion protein. A band was detected using an anti-his antibody and a similar band was detected in the same immunoblot with the dystonin-b antibody (Fig. 1). To ensure that the dystonin-b antibody was not targeting the his-epitope used in its generation, Cos-1 cells were transfected with a flag-tagged IFBD2 mammalian expression fusion plasmid. As anticipated, detection of dystonin-b and the flag-tag revealed overlapping fluorescence signals (Fig. 1). Furthermore, dystonin-b localized in a striated pattern in longitudinal sections of cardiac tissue from wt mice (Fig. 1). This staining pattern was not detected in cardiac tissue sections from *dt* mice processed in parallel (Fig. 1). Immunofluorescence control experiments were conducted to ascertain the specificity of our dystonin-b antibody and to rule out the possibility of non-specific signals. For instance, cardiac muscle sections were stained with either pre-immune serum, primary dystonin-b antibody but no secondary antibody, and with secondary antibody without primary detection. In all instances, immunofluorescence signals were not detected (data not shown).

### Localization of Dystonin in Cardiac Muscle

**Figure 2:**
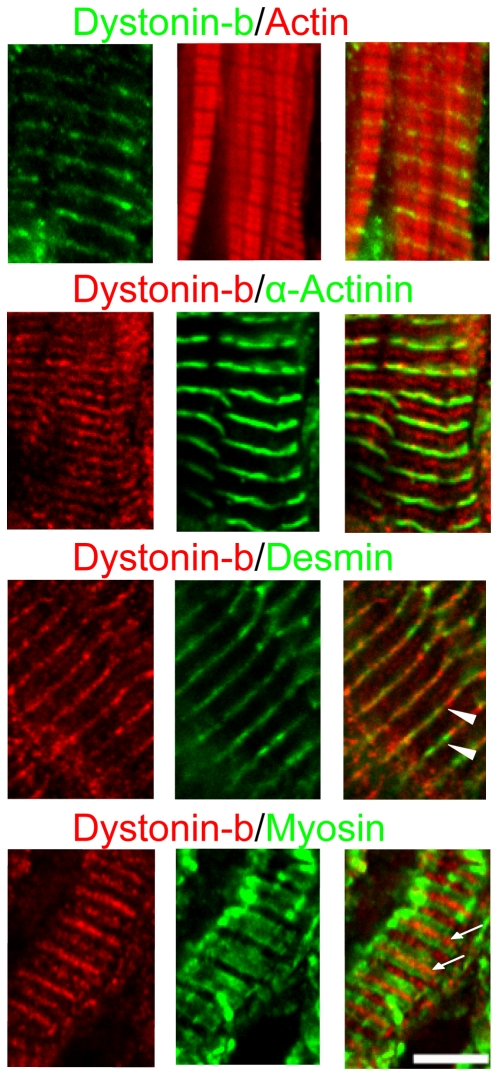
Sarcomeric localization of dystonin-b in cardiac muscle tissue. Longitudinal wild-type cardiac muscle tissue sections were stained with the dystonin-b antibody and other sarcomeric proteins as indicated. The dystonin-b antibody localizes at Z-discs (arrow heads) and at the H zone (arrows). Dystonin-b does not co-distribute with actin or myosin. Scale bar  = 5 µm for all images.

**Figure 3:**
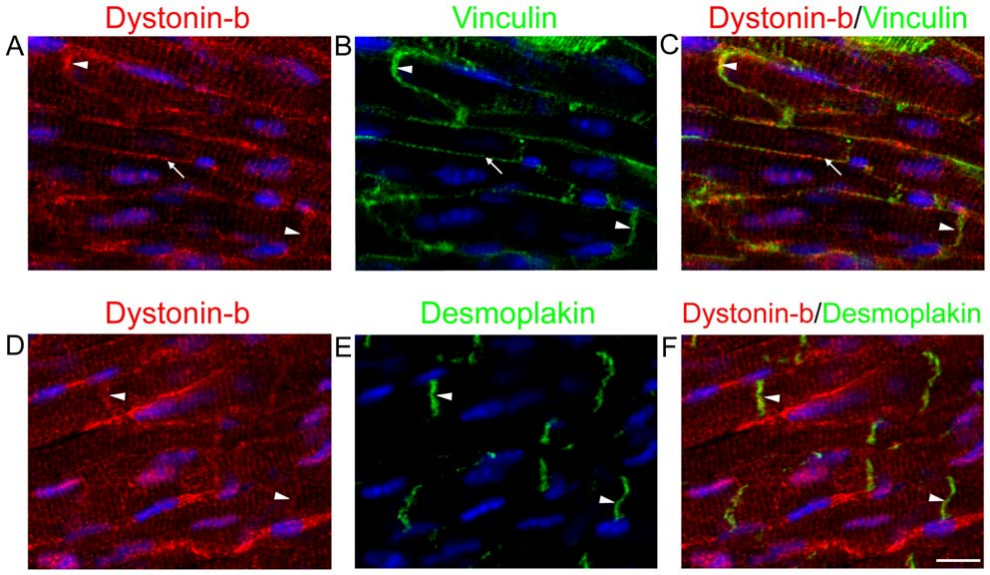
Dystonin-b localizes to the sarcolemma and at intercalated discs. Dystonin-b staining patterns of wild-type cardiac tissue were compared to those of vinculin (B, arrowheads) and desmoplakin (E, arrowheads) both known intercalated disc markers. In addition, vinculin is also found at the sarcolemma (B, arrow). The dystonin-b antibody localizes at the sarcolemma (A, arrow) and at the intercalated disc adhesion structure (A, arrowheads). Scale bar  = 10 µm for all images.

The dystonin-b antibody produced distinct sarcomeric striations in wt cardiac muscle sections (Fig. 2). This antibody stained one distinct band that localized with the Z-discs as well as a second band that was observed between the Z-discs and within the H zone (Fig. 2). Although dystonin-b striations appear to coincide with Z-discs, only partial co-localization of dystonin-b with either desmin or α-actinin was observed. Dystonin-b signal also did not co-localize with either actin or myosin (Fig. 2). Furthermore, dystonin-b antibody localized to the sarcolemma and intercalated discs as revealed by double immunofluorescence studies using vinculin antibody (Fig. 3). The localization of dystonin-b to the intercalated disc adhesion structure was further confirmed by co-staining for desmoplakin (Fig. 3). Finally, we characterized the localization of dystonin-b in H9C2 cardiomyoblasts and found that it had a polarized peri-nuclear distribution in these cells (Figure S2).

### Impact of Dystonin-Deficiency on the Primary Cytoskeletal Networks of the Heart

**Figure 4:**
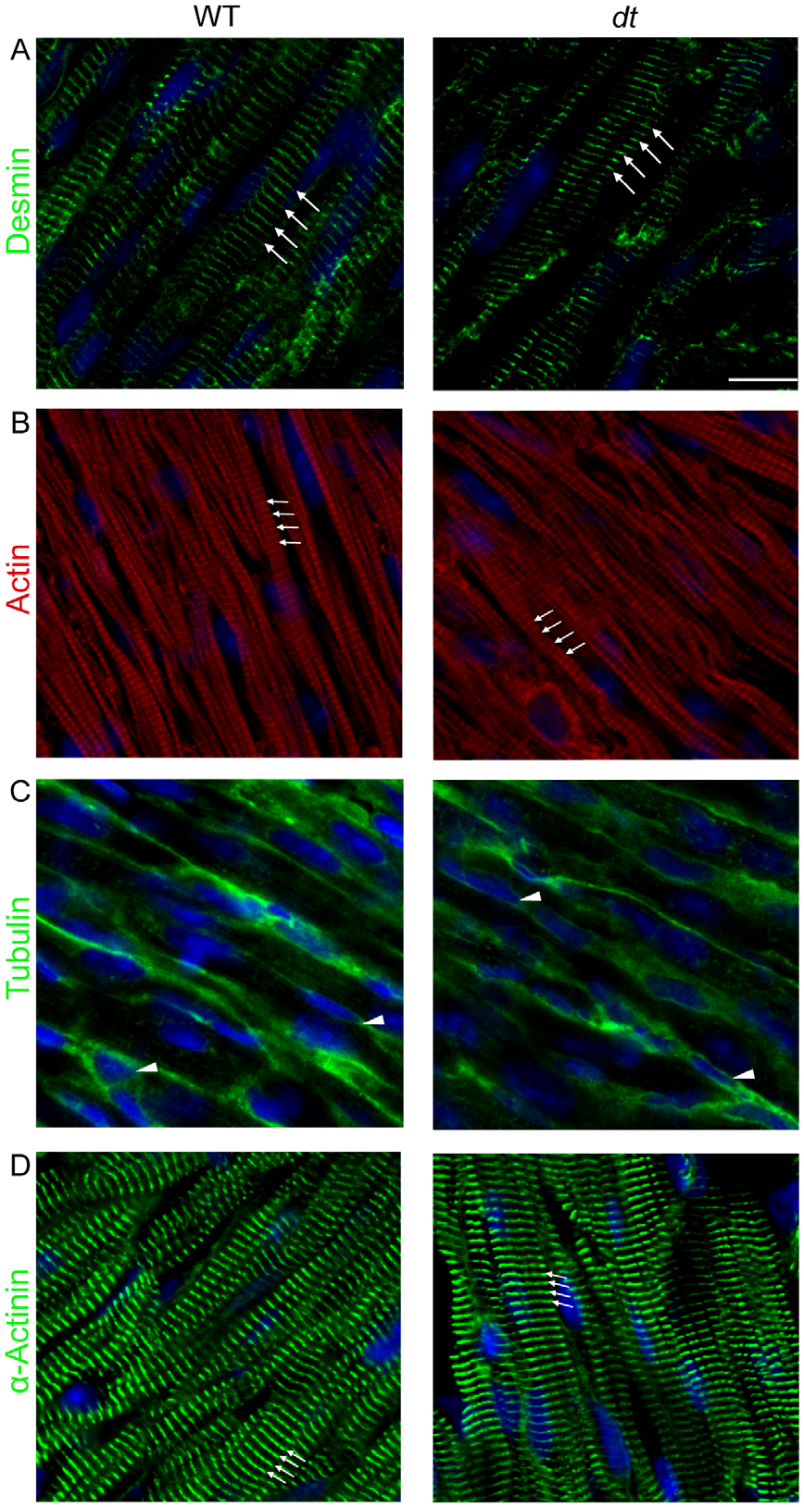
Localization of desmin intermediate filaments, microfilaments, microtubules and α-actinin in longitudinal heart sections from P14 wt and *dt* mice. A total of four wt and four *dt* hearts were processed for immunofluorescence in three separate experiments. Approximately 40 cardiomyocytes were analyzed from one field of view taken from each wt and *dt* sample. (A) Desmin striations and interstriation spacing are comparable in both wt and *dt* samples (arrows). (B) The microfilament striation patterns are also very similar in both wt and *dt* cardiac myocytes. (C) The microtubule network is visible at the perinuclear regions (arrowheads) and near the sarcolemma in both wt and *dt* samples. (D) Alpha-actinin staining was performed to determine the integrity of Z-discs. The staining pattern of α-actinin was similar for both wt and *dt* samples. The nuclei were labeled using DAPI (shown in blue). Scale bar  = 20 µm for all images. N = 4 per group for A–C and N = 3 for D.

Based on the known structure of dystonin isoforms and their localization pattern, we hypothesized that the absence of this protein in *dt* mice would lead to perturbations of the primary cytoskeletal networks, namely the microfilaments, microtubules and desmin intermediate filament systems. Therefore the protein expression levels of actin (all actin isoforms), α-tubulin and desmin as well as the cellular localization of these cytoskeletal components in wt versus *dt* hearts was compared. Western blot analyses revealed that the protein expression levels of these primary cytoskeletal components were unaffected in *dt* hearts compared to wt counterparts (Figure S1). Furthermore, immunofluorescence experiments revealed that the localization of microfilaments, microtubules and desmin intermediate filaments, as assessed by confocal microscopy was similar in *dt* versus wt mice. In particular, distinct striations corresponding to actin (Fig. 4A) and to desmin (Fig. 4B) were observed in cardiac muscle cells from both wt and *dt* samples. Dystonin-deficiency had no effect on the distribution of microtubules at the sarcolemma and within the perinuclear regions of both wt and *dt* muscle fibers (Fig. 4C). To assess whether the structural integrity of the Z-discs was compromised in *dt* mice, we stained for α-actinin, a known Z-disc marker. Alpha-actinin staining revealed Z-discs that were well aligned and evenly spaced in both wt and *dt* samples. (Fig. 4D). The staining pattern for connexin-43 and desmoplakin corresponding to intercalated discs was also comparable in both wt and *dt* heart sections suggesting that these adhesion structures are likely unaltered in dystonin-deficient cardiac muscle (data not shown).

### Assessing the Expression of Markers of Cardiac Hypertrophy in *dt* Hearts

**Figure 5:**
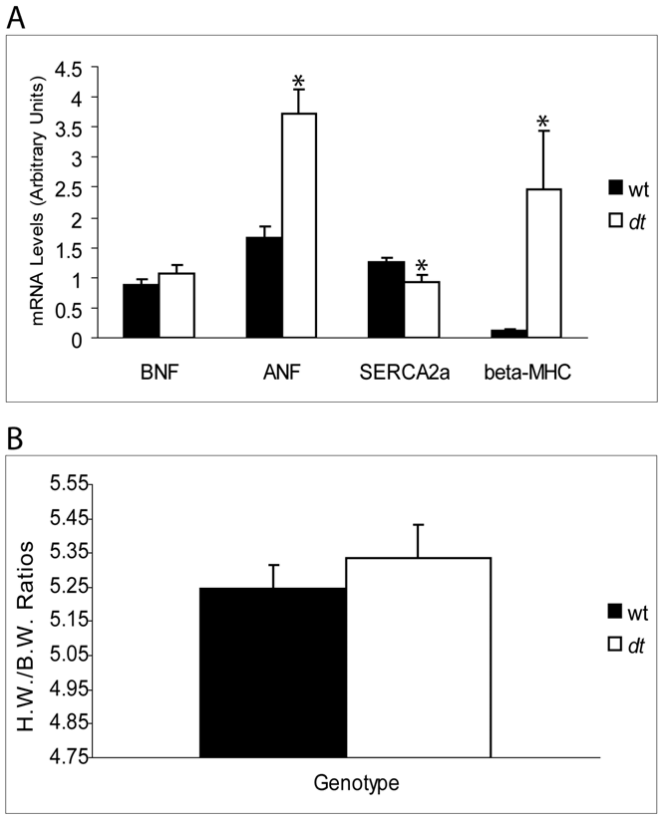
Gene expression analysis of cardiac hypertrophy markers and h.w.(mg)/b.w. (g) ratio comparison in hearts of P14 wt and *dt* mice (data are means ± SEM, N = 10 per genotype for gene expression analysis and N = 16 per group for the ratio data). (A) Compared to control mice, ANF and beta-MHC mRNA levels are elevated and SERCA2a mRNA abundance is lower in *dt* mice. (B) No difference in h.w./b.w. ratio data was observed between wt and *dt* mice. BNF, b-type natriuretic factor; ANF, atrial natriuretic factor; SERCA2a, sarcoplasmic reticulum calcium pump isoform 2a; beta-MHC, β-myosin heavy chain. Asterisks (*) denote significance.

Even though the primary cytoskeletal networks appeared intact, we tested the mRNA expression levels of routinely used cardiac hypertrophy markers such as atrial natriuretic factor (ANF), b-type natriuretic factor (BNF), β-myosin heavy chain sarcoplasmic reticulum calcium pump isoform 2A (SERCA2a) and phospholamban by QPCR. Up-regulation of ANF, BNF, and β-myosin heavy chain as well as down-regulation of SERCA2a and phospholamban were expected as these trends are indicative of hypertrophy [24]. Compared to wt, ANF transcript abundance in *dt* muscle was up-regulated by 66% (*p<0.05*), that of β-myosin heavy chain mRNA levels was increased by 95% (*p<0.05*) while no change in the levels of BNF and phospholamban (data not shown) mRNA was detected (*p>0.05*) (Fig. 5A). Conversely, a 26% decrease in the levels of SERCA2a transcripts was observed in *dt* hearts compared to wt (*p<0.05*). Taken together, this expression profile suggests possible early molecular signs of cardiac tissue abnormalities in *dt* mice.

### Impact of Dystonin-Deficiency on Morphological and Histological Parameters of Heart Tissue

**Figure 6:**
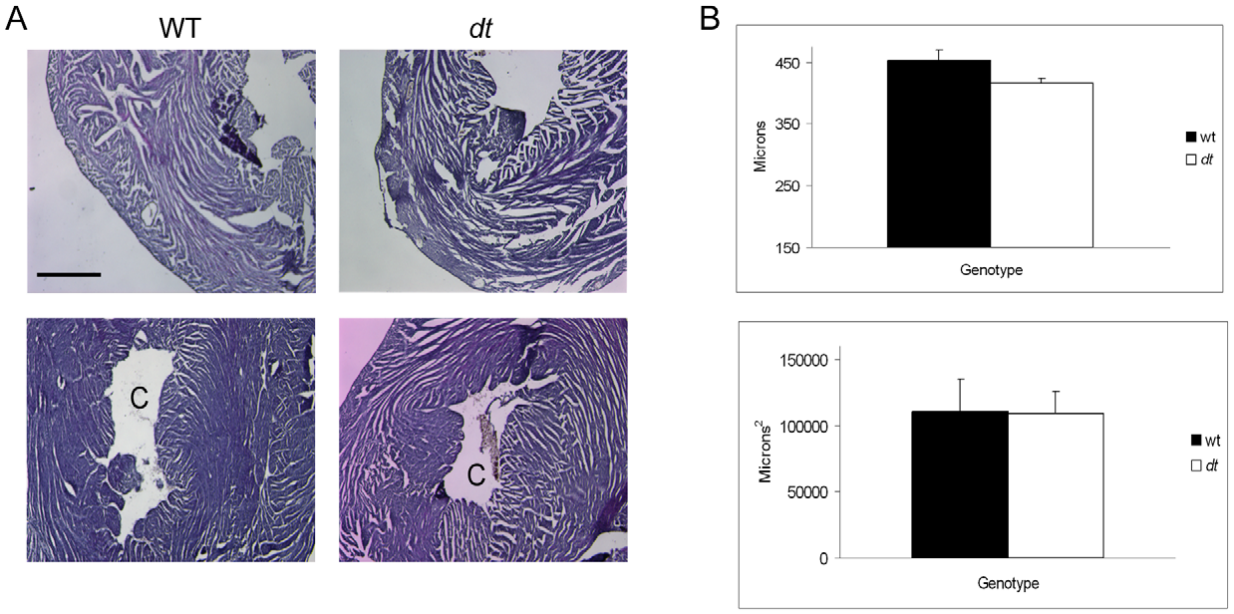
Histological analysis of the left ventricle wall thickness and chamber area of wt and *dt* hearts. (A) Hematoxylin and eosin-stained cross sections of hearts obtained from wt and *dt* mice. The upper panels (A) show the left ventricle thickness and the lower panels represent the left ventricle chamber (area denoted with a C). (B) Left ventricle wall thickness data (top graph) and chamber area data (lower graph) obtained from wt and *dt* mice (mean ± SEM, n = 9 per group). Five wall thickness measurements were collected for each heart sample then averaged. Scale bar  = 100 µm for all images.

**Figure 7:**
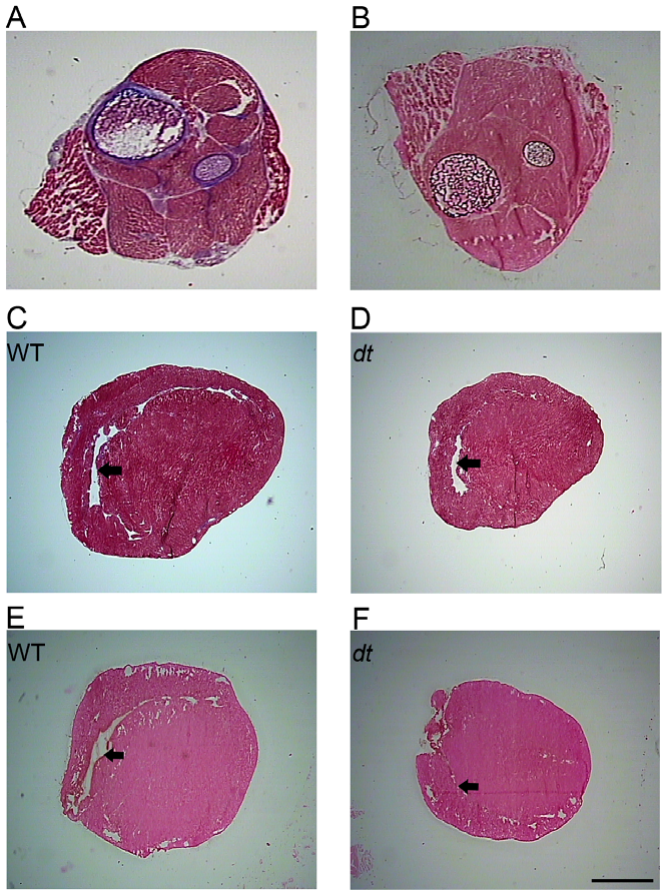
Masson Trichrome and Von Kossa-stained wt and *dt* longitudinal heart sections to visualize fibrosis and calcium deposits respectively (N = 6). A and B are wt whole hindlimb which include muscle and bone (positive control). (A) Connective tissue surrounding the bones and the muscle (endomysium, perimysium and epimysium) is stained blue. (C) and (D) are heart muscle sections from wt and *dt* samples respectively showing no signs of fibrosis (the black arrows in C, E, F and D denote the right ventricles and the apex is pointed toward the right side). (B) The black staining present in the bones demonstrates the presence of calcium. No calcium deposits were observed in heart muscle sections from wt (E) and *dt* samples (F). Scale bar in F = 1000 µm is applicable for all images.

Based on the results from our molecular analysis of hypertrophic markers, we hypothesized that the deficiency of dystonin in *dt^Tg4^* mice would lead to signs of cardiomyopathy at morphological and histological levels. Though the average h.w./b.w. ratios tended to be higher in *dt* mice the differences between wt and *dt* groups were not statistically significant (*p>0.05*) (Fig. 5B). Assessment of the left ventricle wall thickness and left ventricle chamber area for the wt and *dt* mice showed no significant difference between the two groups (*p>0.05*, Fig. 6). Finally, signs of fibrosis and calcification were not observed in *dt* heart sections (Fig. 7). Together, these results suggest that the absence of dystonin has no overt effects on the morphological and histological features of the heart, at least in young postnatal mice.

### Expression of Other Cardiac Muscle Plakins in Dystonin-Deficient Hearts

**Figure 8:**
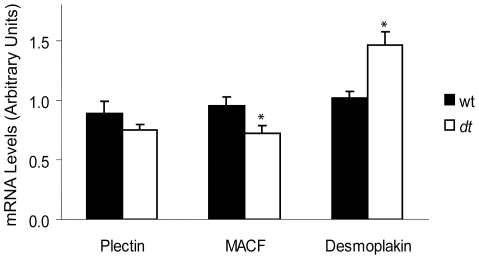
Gene expression analysis of plectin, MACF and desmoplakin in wt versus *dt* hearts (data are means ± SEM, n = 10 per group). mRNA was isolated from wt and *dt* hearts and QPCR was performed to assess transcript levels of other plakin proteins expressed in cardiac tissue.

Plectin, MACF and desmoplakin are other plakin family members expressed in cardiac tissue that share several sequence similarities with dystonin [1], [9], [25], [26]. Given that the morphological and histological parameters as presented above are largely unchanged in *dt* hearts, we hypothesized that the expression levels of plectin, MACF and desmoplakin may be up-regulated to compensate for the lack of dystonin in *dt* hearts. The transcript levels of plectin, MACF and desmoplakin in wt versus *dt* hearts were therefore measured by QPCR (Fig. 8). Only desmoplakin mRNA levels were up-regulated (44%) in the hearts of *dt* mice (*p<0.05*). No change was detected in the levels of plectin mRNA (*p>0.05*), while a 25% reduction in MACF transcript levels was observed (*p<0.05*).

## Discussion

To investigate the effects of dystonin-deficiency on cardiac muscle, we identified the sarcomeric localization of dystonin isoform-b, the predominant muscle isoform of dystonin in cardiac tissue. Our results show that the primary cytoskeletal components, namely microfilaments, desmin intermediate filaments, and microtubules remain intact in *dt* hearts. Interestingly, dystonin-deficient hearts do display features of hypertrophic cardiomyopathy at the molecular level but not at the morphological level.

### Dystonin-b Localization in Cardiac Muscle

We have generated an antibody to target the muscle isoform of dystonin. Our immunofluorescence data suggest that dystonin-b is localized at two specific sites in cardiac muscle fibers, namely at the Z-disc and within the H zone. The dystonin-b antibody produced a strong signal at Z-discs similar to desmin and α-actinin. As compromised Z-disc ultrastructure has been previously reported in skeletal muscle from *dt* mice [27], it is possible that dystonin may play a role in assuring the structural integrity of Z-discs [21], [23]. At present, the role of dystonin-b at the Z-disc is not clear. Based on our immunofluorescence studies, it is unlikely that dystonin directly stabilizes desmin intermediate filaments at the Z-disc since the localization of desmin remains intact in *dt* hearts.

A second striation detected with the dystonin-b antibody was parallel to the Z-disc and appeared to be present in the H-zone. Within this region, dystonin-b may further strengthen the sarcomere and protect it from contraction-induced stress along with other proteins such as titin [28]. In addition, our findings demonstrate that dystonin-b is found at the sarcolemma and intercalated discs in mouse cardiac muscle. Intercalated discs are composed of other plakin family members such as plectin and desmoplakin. The requirement for multiple cytolinker proteins at this cardiac muscle adhesion structure likely reflects the greater need for structural support to resist the effects of chronic mechanical stress. Three N-terminal variants of dystonin-b isoforms, namely isoforms b1, b2 and b3 are expressed in cardiac tissue (Boyer and Boudreau-Larivière, unpublished[16]) though their individual distribution in cardiac muscle fibers is not presently known. These unique N-terminal regions have been shown to direct dystonin fusion proteins to distinct compartments within cells maintained in culture [14], [15], [16]. Our dystonin-b antibody should recognize all three muscle isoforms since it is targeted to the muscle isoform-specific IFBD2 region. It is therefore reasonable to anticipate that the dystonin signals detected near Z-discs, the H zone, intercalated discs and the sarcolemma may correspond to distinct N-terminal variants of the dystonin-b isoforms. Plectin isoforms harboring unique sequences within the N-terminal region accumulate preferentially within specific regions of the muscle fiber including the Z-disc (plectin 1d), the costameres (plectin 1/1f), and in close proximity to mitochondria (plectin 1b) [3], [5], [29]. A similar scenario is likely to emerge for dystonin-b isoforms.

### Dystonin-Deficiency in Mice Does Not Significantly Affect the Primary Cytoskeletal Components

Given the suggested localization of the dystonin isoforms [13], [14], [15], [27] and the fact that these variants harbor interacting domains for microfilaments, intermediate filaments and microtubules, it was expected that these filament systems may be destabilized in dystonin-deficient cardiac tissue. Our protein analyses reveal that desmin, actin and α-tubulin protein levels are largely unaltered in *dt* samples, and that the absence of dystonin in cardiac tissue has little effect on the subcellular localization of the primary cytoskeletal elements in tissues from two-week old animals. Although, it was difficult to obtain high-resolution immunofluorescence images of microtubules, the tubulin staining pattern observed was very similar between wt and *dt* samples. Taken together with the unchanged tubulin protein levels, we conclude that the microtubule network is likely not perturbed in dystonin-deficient cardiac muscle. However, we cannot rule out the possibility that the composition of microtubules, for instance changes in acetylated or glutamylated tubulin may be occurring in dystonin-deficient cardiac muscle. Both wt and *dt* samples displayed well defined striations corresponding to either actin microfilaments or desmin intermediate filaments. These findings are contrary to what we had hypothesized, based on co-localization studies [14], [21] suggesting a direct or indirect link of dystonin with the microfilament and desmin intermediate filament cytoskeletal elements in muscle. Desmin is an unlikely candidate to bind to the IFBD2 region of the dystonin-b isoform because the desmin network appears to be well preserved in *dt* hearts. In the myocardium, loss of plectin in mice leads to disorganized sarcomeric structures, disintegrated intercalated discs and death within the first two days of post-natal development [30]. Recent data obtained using a muscle-specific plectin-null mouse model suggests that plectin is the primary desmin organizer in muscle [3]. In *dt* hearts, plectin mRNA expression (and likely protein levels) remains relatively stable and appears to be sufficient to preserve desmin myocardial structure. It is therefore possible that other intermediate filaments present in cardiac muscle including synemin, paranemin, and cytokeratins, could be the primary intermediate filaments associated with dystonin-b. Future studies should focus on this aspect to better understand the contributions, if any, of dystonin in stabilizing intermediate filament networks.

Levels of mRNA encoding desmoplakin were elevated in *dt* versus wt hearts while MACF transcript abundance was decreased. Desmoplakin binds desmin intermediate filaments [31] at the dense, inner plaque of the desmosome in cardiac muscle intercalated discs effectively regulating the adhesive strength necessary for maintaining tissue integrity [32]. The mild increase in desmoplakin expression in *dt* hearts may signal an attempt by cardiac muscle fibers to compensate for the lack of dystonin. This may explain why intercalated discs are intact in dystonin-deficient hearts. More specifically, our immunolocalization data would suggest that despite being present at intercalated discs, dystonin is not likely a key stabilizer of these structures given that desmoplakin and connexion-43 staining appear largely unaltered in dystonin-deficient hearts. A MACF isoform has been shown to harbor a putative IFBD in the center of the protein similar to that described for dystonin-b [33]. If abundantly expressed in heart muscle, this MACF isoform may meet all the structural criteria needed to compensate for the absence of dystonin, however, the results indicate a moderate decrease in MACF expression in *dt* hearts at least at the mRNA level. Taken together, we predict that desmoplakin may be the primary desmin IFs stabilizer at intercalated discs and that plectin is the primary desmin organizer within the sarcomere. The precise architectural functions of dystonin and MACF muscle isoforms remains to be clearly defined.

### *dt* Mice Display Signs of Cardiomyopathy at the Molecular Level but Not at the Histological and Morphological Levels

Although our findings reveal no overt morphological indicators of cardiac hypertrophy in *dt* hearts from 2-week old mice, our gene expression analysis of ANF (upregulated), BNF (unchanged) and SERCA2a (downregulated) would suggest mild cardiac stress in *dt* hearts from young animals. For instance, in cases of severe cardiomyopathy such as heart failure, ANF levels are usually up-regulated 5–10 times in diseased hearts compared to controls [34], [35]. In the present study, a 2.2 fold increase in ANF expression was observed in *dt* hearts. In comparison,we detected a 20 fold increase in β-myosin heavy chain mRNA levels representing an up-regulation that is well over that observed in rodent models of cardiac hypertrophy [36].

One possible source of the stress can be linked to a weakened diaphragm muscle. Diaphragm muscles from *dt* mice generate less force, fatigue quicker and are more susceptible to mechanically-induced stress compared to wt diaphragm muscles [21]. Given the importance of this muscle in breathing, a compromised diaphragm muscle in *dt* mice could result in inefficient oxygen intake leading to compensatory mechanisms from the cardiovascular system to maintain suitable oxygen saturation levels.

### Why Do *dt* Mice Die before the Age of Weaning?

*Dystonia musculorum* mice die before the age of weaning of unknown causes. It is unlikely that premature death of *dt* mice can be attributed to an intrinsic cardiomyopathy because this investigation revealed that *dt* hearts do not display overt signs of morphological or histological cardiac defects. Furthermore, according to the immunofluorescence experiments, the distribution of the primary cytoskeletal networks remains relatively intact when dystonin is absent. A plausible reason for the premature death of the *dt* mice could therefore be related to a compromised diaphragm muscle [21] that would hinder the breathing capacities of *dt* mice.

It is not known if the lack of one dystonin isoform in particular is responsible for the *dt* condition or if the absence of multiple isoforms is the underlying cause. Determination of the dystonin variant that is responsible for the disease may help us identify the precise cause of death in these mice. Rescue studies as well as tissue and isoform specific conditional knock-out experiments like the ones performed for plectin isoforms [3], [29] have been proposed to investigate whether one or multiple isoforms are responsible for the *dt* disorder [7]. Additional studies targeting specific tissues, such as muscle, in which dystonin is expressed, are needed to further elucidate the underlying pathophysiology of the *dt* disease. For instance, cardiac muscle-specific dystonin knockout mice would be useful to investigate whether they would develop overt morphological defects of the heart with age or whether the absence of dystonin correlates with compromised exercise capacity.

In summary, we reveal that dystonin-b isoforms localize at the Z-disc, within the H zone and the sarcolemma of cardiomyocytes as well at intercalated discs of cardiac tissue. Our results show that dystonin-deficiency does not lead to overt cardiac defects at least in young mice. However, the expression profile of the hypertrophic markers in *dt* hearts is an early indicator of cardiac stress that may be related to extrinsic factors. Whether dystonin-deficiency leads to an intrinsic cardiac muscle defect in the long term is for the moment, not known.

## Supporting Information

Supplemental information S1 (0.03 MB DOC)

Figure S1 Desmin, α-tubulin and actin protein levels in hearts from P14 wt and dt mice as determined by western blot analysis. (A) Representative example of SDS-PAGE western blot results and coomassie-stained membrane demonstrating equal protein loading. (B) Densitometric analysis of western blots. Band densities were determined by image analysis and normalized to the sum of the band densities from the coomassie-stained membrane (data are means ± SEM, N = 8 per group).
(2.08 MB TIF)

Figure S2 Endogenous signal detection of dystonin-b antibody in H9C2 cardiomyoblasts. (A) The dystonin-b antibody (red) appears to stain the perinuclear region in H9C2 cardiomyoblasts with signal aggregation to one side of the nucleus (blue). (B) Staining for the Golgi marker, Trans-Golgi network-38, revealed a strong polarized signal which co-localized with dystonin-b (C). Nuclei are visualized in blue with DAPI nuclear stain. Scale bar  = 10 µm.
(9.80 MB TIF)
