# Supplementary material for: Hearts of Dystonia musculorum Mice Display Normal Morphological and Histological Features but Show Signs of Cardiac Stress
Source: PLoS One. 2010 Mar 1;5(3):e9465. doi: 10.1371/journal.pone.0009465 (PMC2830884; doi:10.1371/journal.pone.0009465)
Supplement: Supplemental information S1 — (0.03 MB DOC) [file pone.0009465.s001.doc]

**Supplemental Information S1**

**Supplemental Material and Methods**

*Protein expression - Bacterial*

BL-21 *Escherichia coli* were transformed with the appropriate plasmid and streaked on a Luria’s broth (LB; 1% bacto-tryptone, 1% NaCl, 0.5% bacto-yeast) plate containing antibiotics for selection. Plates were incubated at 37°C overnight. Colonies were picked the next day and grown in culture tubes containing LB and the appropriate antibiotic overnight at 37°C in a shaking incubator. The overnight culture (0.5 ml) was added to a 500 ml Erlenmeyer flask containing 100 ml of LB with antibiotics and incubated at 37°C for 3 hours while agitated. To induce protein expression, isopropyl β-D-1-thiogalactopyranoside (IPTG) was added to the culture flask, which was then incubated at 32°C for 4 hours. The bacterial culture was centrifuged at 9000 RPM for 10 minutes at 4°C. The supernatant was removed and the pellet was resuspended in STE (0.1 M NaCl, 10 mM Tris-HCl, 1 mM EDTA) containing 1.5% N-lauroyl sarkosyl. Resuspended pellets were sonicated (Sonics and Materials Inc., Connecticut) for 2 minutes at 35% amplitude with a 1 second on/off pulse while kept on ice. Triton X-100 (0.1%) was added to the sonicated lysate which was centrifuged at 13000 RPM for 5 minutes at 4°C. Soluble (supernatant) and insoluble (pellet) fractions were separated and either used immediately or stored at -80°C for further analysis.

**Supplemental Results**

*Dystonin-b Localizes in a Perinuclear Fashion in H9C2 Cardiomyoblasts*

We characterized the localization pattern of the antibody in H9C2 cardiomyoblast cells. A distinct staining pattern was observed with polarized perinuclear aggregation of the signal to one side of the nucleus (Fig. S2A). To elucidate the nature of this staining pattern, immunofluorescence studies were performed using antibodies targeting sub-cellular markers. The dystonin-b antibody produced a robust signal that associates with a trans-Golgi network marker (TGN-38) but not to the endoplasmic reticulum marker protein disulfide isomerase (data not shown). These results support our previous findings showing dystonin associates to the Golgi in C2C12 myoblasts [1]. Furthermore, MACF1b a plakin sharing high homology with dystonin-b also targets the Golgi [2]. For the moment, the role of plakin family members at the Golgi is unclear but may include ensuring proper Golgi localization [3].

Supplemental Information References

1. Young KG, Pinheiro B, Kothary R (2006) A Bpag1 isoform involved in cytoskeletal organization surrounding the nucleus. Exp Cell Res 312: 121-134.

2. Lin CM, Chen HJ, Leung CL, Parry DA, Liem RK (2005) Microtubule actin crosslinking factor 1b: a novel plakin that localizes to the Golgi complex. J Cell Sci 118: 3727-3738.

3. Boyer JG, Bernstein MA, Boudreau-Lariviere C (2009) Plakins in striated muscle. Muscle Nerve.
