# Supplementary figures and images for: Hearts of Dystonia musculorum Mice Display Normal Morphological and Histological Features but Show Signs of Cardiac Stress
Source: PLoS One. 2010 Mar 1;5(3):e9465. doi: 10.1371/journal.pone.0009465 (PMC2830884; doi:10.1371/journal.pone.0009465)

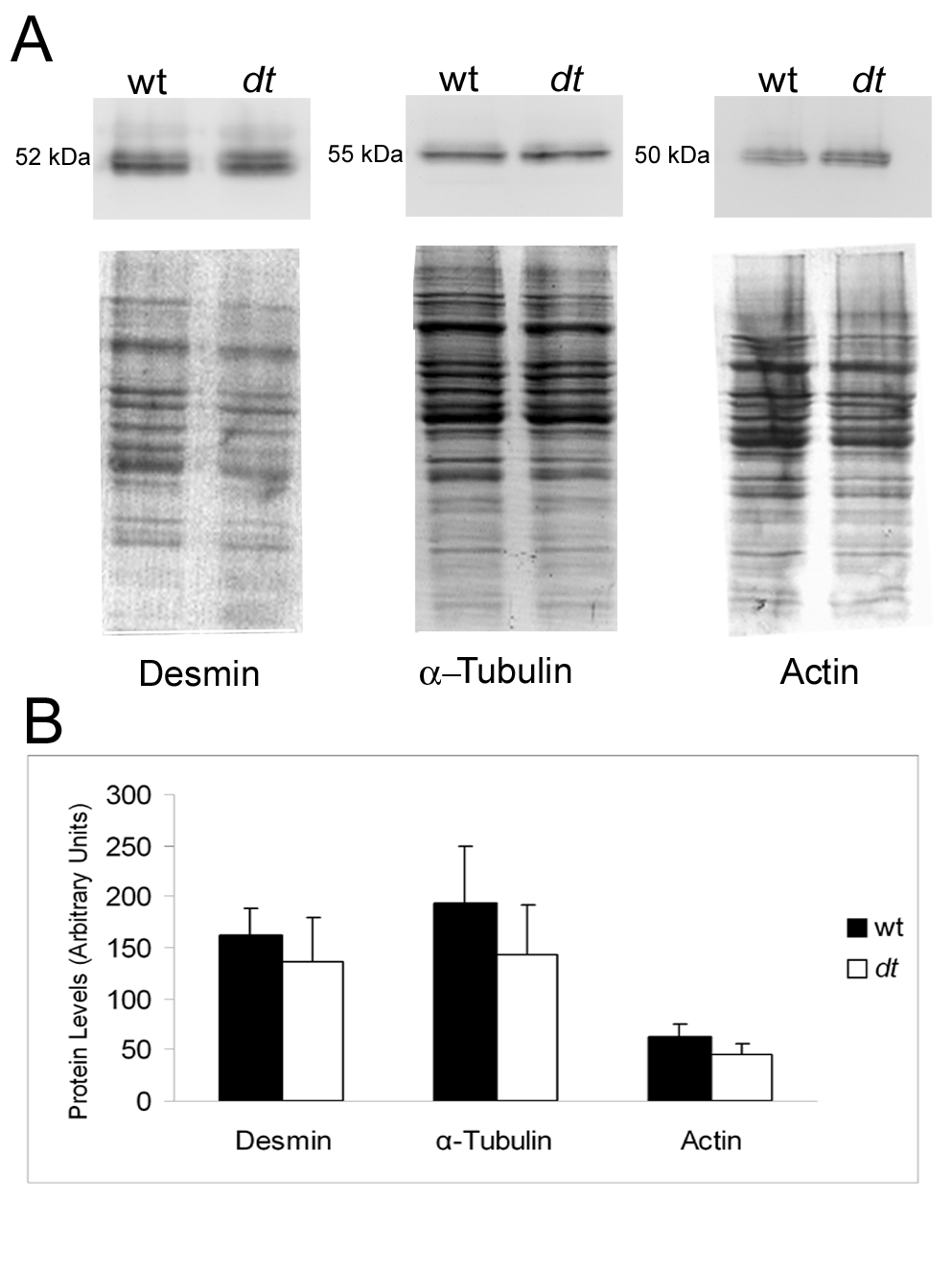

Supplement: Figure S1 — Desmin, α-tubulin and actin protein levels in hearts from P14 wt and dt mice as determined by western blot analysis. (A) Representative example of SDS-PAGE western blot results and coomassie-stained membrane demonstrating equal protein loading. (B) Densitometric analysis of western blots. Band densities were determined by image analysis and normalized to the sum of the band densities from the coomassie-stained membrane (data are means ± SEM, N = 8 per group). (2.08 MB TIF) [file pone.0009465.s002.tif]

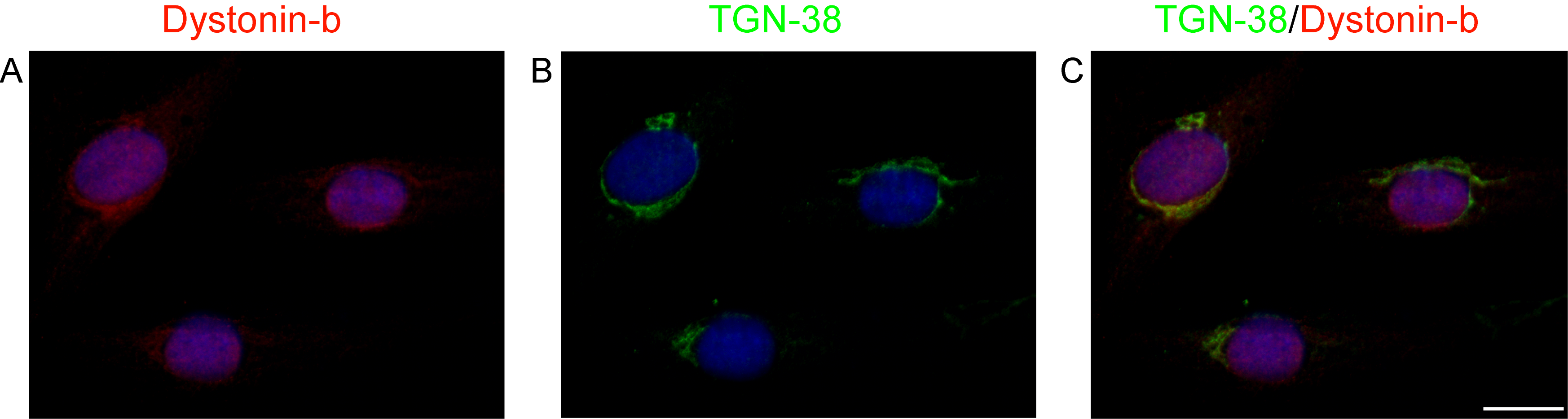

Supplement: Figure S2 — Endogenous signal detection of dystonin-b antibody in H9C2 cardiomyoblasts. (A) The dystonin-b antibody (red) appears to stain the perinuclear region in H9C2 cardiomyoblasts with signal aggregation to one side of the nucleus (blue). (B) Staining for the Golgi marker, Trans-Golgi network-38, revealed a strong polarized signal which co-localized with dystonin-b (C). Nuclei are visualized in blue with DAPI nuclear stain. Scale bar = 10 µm. (9.80 MB TIF) [file pone.0009465.s003.tif]
